# Physical and Sensory Properties of Japanese Quince Chips Obtained by Osmotic Dehydration in Fruit Juice Concentrates and Hybrid Drying

**DOI:** 10.3390/molecules25235504

**Published:** 2020-11-24

**Authors:** Hanna Kowalska, Agata Marzec, Ewa Domian, Ewelina Masiarz, Agnieszka Ciurzyńska, Sabina Galus, Aleksandra Małkiewicz, Andrzej Lenart, Jolanta Kowalska

**Affiliations:** 1 Department of Food Engineering and Process Management, Institute of Food Sciences, Warsaw University of Life Sciences, 159c Nowoursynowska St., 02-776 Warsaw, Poland; agata_marzec@sggw.edu.pl (A.M.); ewa_domian@sggw.edu.pl (E.D.); ewelina_masiarz@sggw.edu.pl (E.M.); agnieszka_ciurzynska@sggw.edu.pl (A.C.); sabina_galus@sggw.edu.pl (S.G.); malkiewicz.aleksandra94@gmail.com (A.M.); andrzej_lenart@sggw.edu.pl (A.L.); 2 Department of Technology and Food Evaluation, Institute of Food Sciences, Warsaw University of Life Sciences, 159c Nowoursynowska St., 02-776 Warsaw, Poland; jolanta_kowalska@sggw.edu.pl

**Keywords:** fruit chips, osmotic treatment, convection and microwave-vacuum drying (hybrid), freeze-drying, mechanical and acoustic properties, PCA

## Abstract

Japanese quince has high health value, but due to its taste and texture, it is difficult to eat raw. The use of innovative drying methods to produce dried snack foods from these fruits may be of interest to producers and consumers. The physicochemical and sensory properties of 3 mm slices of Japanese quince fruit (with skin, without seeds) obtained by osmotic pre-treatment in chokeberry and apple juice concentrates, and with the use of convection (convective drying, C-D), freeze-drying (F-D), and convection-microwave-vacuum drying (hybrid) are assessed. The methods of drying osmo-dehydrated slices do not affect the dry matter content. In most dried quince, the water activity is 0.40 or lower. Pre-osmotic dehydration and drying have a significant impact on the mechanical and acoustic properties of quince chips. Sensory attractive chips emit loud acoustic emission (AE) during the breaking test. Chips that are osmo-dehydrated in a mixture of chokeberry juice concentrate and sucrose and dried by a hybrid method are attractive. They have a dark red color given by chokeberry concentrate and a slight sweet (with a slight sour-bitter) taste. The sensory evaluation was useful for determining the quality of the chips in terms of their texture (crispness) tested by mechanical methods. Their sensory ratings (overall desirability as weight of color, taste, crispness, and flavor) are high and similar (from 3.8 to 4.1). The use of innovative drying methods with pre-osmotic treatment allows obtaining dried material with properties comparable to those obtained by the F-D method, but in a much shorter time, i.e., with lower energy and using a simple method.

## 1. Introduction

Food producers are trying to respond to current challenges and develop new concepts of products with high pro-health values. Farzaneh et al. [1] highlighted the growing interest in different plants with pro-healthy properties for different purposes compared to synthetic additives and preservatives. The mechanism of action of natural bioactive compounds with health benefits was extensively described by Farzaneh and Carvalho [2], and the various techniques to improve the extraction of bioactive ingredients and their properties (antioxidant, anticarcinogenic, antimicrobial, and antidiabetic) were explained by Farzaneh and Carvalho [3], Moghimi et al. [4]. The use of fruits, such as quince or chokeberry, which are unpopular due to their bitter, tart taste, or for being too hard, but yet, have high contents of pro-health ingredients, may create new forms of products, e.g., by giving them color or a unique taste [5]. Antoniewska et al. [6] added freeze-dried Japanese quince fruit to cookies to enrich and increase their sensory characteristics.

Japanese quince fruit (*Chaenomeles japonica*) is a common species occurring in Poland, but mainly as a garden decoration. The chemical composition studies of Japanese quince fruit (quince fruit) reported the presence of many biologically active compounds, such as polyphenolics, organic acids, terpenoids, alcohols, ketones, and aldehydes. The fruit is low in sugar and rich in dietary fiber (28–38 g/100 g) [7,8,9]. The fruit is characterized by overall acidity in the range of 3.9–4.1%; the ratio of the sugars to acids makes it difficult to consume when unprocessed [8]. It is also characterized by strong antioxidant activity (10,512 µM Trolox/100 g, respectively) and polyphenol content (924 mg of catechin/100 g) [8].

Quince fruit has high nutritional application potential due to its high concentration of vitamin C and its ~20 polyphenolic compounds [10,11,12,13]. In many publications, the consumption of quince fruit is recommended for the prevention or even treatment of various diseases [12,14]. Its anti-inflammatory, analgesic, antispasmodic, antioxidant, immunoregulatory, antibacterial, and antinociceptive properties have been confirmed. It also protects the liver and is used in Parkinson’s disease [15]. However, the quince fruit is characterized by hardness and high acidity, so is unsuitable for direct consumption. It is an appropriate raw material for processing in the food industry, especially by microwave-vacuum drying [7,16]. Quince fruit was shown to inhibit browning of freshly cut pear slices during storage for 9 days [17].

To respond to current challenges regarding the use of little appreciated fruit, such as quince, food producers can use osmotic dehydration (OD) as a pretreatment, which is a well-known and long-used process that is still being improved today [5,18]. The use of fruit or vegetable juices creates new forms of products, e.g., by giving them a different color or unique taste. Crispy (crunchy) snacks that break relatively easily and produce a sharp sound are especially desirable [19]. Lech et al. [20] examined the OD of pumpkin slices in chokeberry, flowering quince, and raspberry concentrated juices. Next, osmo-dehydrated pumpkin was subjected to vacuum-microwave finish drying. Osmotic pre-treatment resulted in a decrease in color coordinates and an improvement in the antioxidant activity of the dried product. These snacks provide an alternative to snacks with low nutritional value.

Fruit or vegetable snacks in the form of chips are often produced by drying [21]. These types of snacks do not require preservation with chemical substances. During drying, the moisture content of the raw material decreases, which leads to the reduction in the water activity, thereby resulting in high product safety. The most commonly used C-D has some disadvantages, so it is often supported by other techniques, such as microwave, infrared, or ultrasound [20,22]. The material is exposed to long-term (several hours or more to obtain a moisture content of around 20%) high temperature of 40–70 °C [22] and oxygen, which causes damage, both optically (shrinkage) and sensory, causing changes in taste, color, and flavor by reducing volatile compounds. Due to the long process time of C-D, this method is energy-intensive and causes the loss of vitamins, polyphenols, and other thermolabile compounds [23]. F-D is the method most suitable for maintaining the nutritional value and quality of a product. Under these conditions, drying involves the sublimation of ice of previously frozen material. Numerous chemical and biological reactions are inhibited. However, the final drying phase is performed at a positive temperature. Incomplete sublimation of ice can lead to deterioration of the quality of the dried material, its shrinkage, and chemical changes [24].

Among the many snack production technologies, the most effective technique is drying fruit using a hybrid method, i.e., microwave vacuum in combination with initial convection drying. It is famous for its unique advantages, mainly due to significant shortening of drying and a high-quality dried product [25]. The sudden increase in temperature and reduced pressure cause evaporation and expansion of the moisture removed from the cells of the material. The process involves the expansion of vapor or gas in the material and, consequently, creates a characteristic internal structure [26,27]. Puffing also has a positive effect on sensory impressions such as the color, taste, smell, and texture of dried fruit [28]. Among the sensory features of snacks in the form of chips, crispness and crunchiness of dried fruit are particularly important, which can both be creating using both the F-D and puffing methods [29].

Chips are made of various fruit (apples, pears, mangoes, peaches, strawberries, and bananas). Unfortunately, the price of the raw material and the technology of producing this type of snacks make them expensive and often inaccessible to all consumer groups. The food industry is looking for a technology that would allow cost optimization by reducing the drying time and increasing production efficiency and product quality. This innovative method allows the use of fruit juice concentrates in the initial osmotic treatment to enrich snacks with a number of natural health-promoting ingredients, and the less popular microwave-vacuum drying method may be of interest to food producers. This solution may provide significant benefits in terms of reducing the time (energy savings) of obtaining high-quality dried fruit with consumer-acceptable characteristics.

The aim of this study was to develop quince fruit chips using osmotically dehydration in fruit juice concentrates and drying using an innovative convection-microwave-vacuum (hybrid) method compared to convection and freeze-drying. The main objective was to determine the physicochemical and sensory properties of dried (crisps) to obtain the optimal texture of dried quince slices.

## 2. Results and Discussion

### 2.1. Osmotic Pre-Treatment

**Figure 1:**
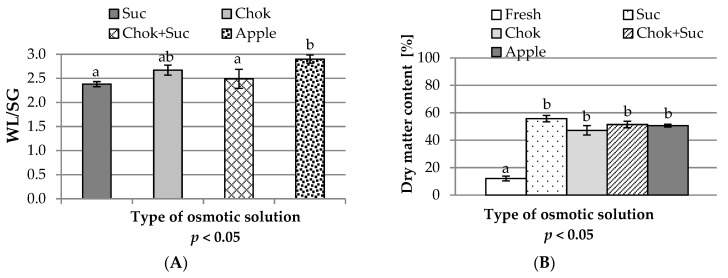
Influence of the type of osmotic solution on: (**A**) dehydration efficiency factor (water loss to solid gain (WL/SG) ratio) and (**B**) dry matter content of osmo-dehydrated (at 50 °C for 24 h) quince slices. Designations: a, b—homogeneous groups; sucrose solution (Suc), chokeberry juice (Chok), Chok + Suc, apple—coding the type of osmotic solution, as given in the experimental procedures.

To assess the effectiveness of OD, the ratio of water loss (WL) to solid gain (SG)—(WL/SG)—of dehydrated fruit slices was determined [21]. It was observed that the higher the value, the greater the dehydration effect compared to the penetration of osmotic substances into the quince fruit samples. The WL/SG coefficients varied significantly and ranged from about 2.38 in the osmo-dehydrated in sucrose solution samples to about 2.9 when apple juice concentrate was used (Figure 1A). The water activity of the solutions had no effect as their values were similar (0.81 ± 0.02), so the molecular mass of the osmotic substances should be considered. The dehydration effect, when using solutions containing low molecular weight substances, is stronger [30]. Concentrates of apple juice, as well as those of chokeberry fruit, contain different water-soluble substances with a lower or higher molecular mass than the sucrose solution. Lech et al. [31] showed that osmotically dehydrated beetroot slices in chokeberry juice did not contain as much sugar as the sucrose solution; therefore, the ingredients of chokeberry juice easily penetrated into the beet tissue. However, in this study, the type of osmotic medium had no significant effect on dry matter content (Figure 1B). Apple and chokeberry concentrated juices are a good medium for OD of plant raw materials, even better than commonly used sucrose because using any solution other than sucrose results in a higher WL/SG ratio. However, this index may be constant when both WL and SG values change in the same way, or WL may increase as SG decreases [32]. In this study, lower WL/SG values in fruit dehydrated in sucrose solution resulted from greater penetration of osmotic substance; therefore, the dry matter content in these samples was the highest (Figure 1B).

The water activity of the fresh Japanese quince fruit was found to be 0.988. As a result of OD, there was a partial replacement of water loss with an osmotic substance, which reduced the water activity of the quince fruit to 0.85–0.88 (11–14% lower). According to Matuska et al. [33], for most pathogenic bacteria, water activity below 0.95 prevents their development. However, OD does not provide the product with complete stability. Further dehydration of the product is required, e.g., by drying.

### 2.2. Properties of Dried Quince Fruit (Chips)

#### 2.2.1. Water Activity

**Figure 2:**
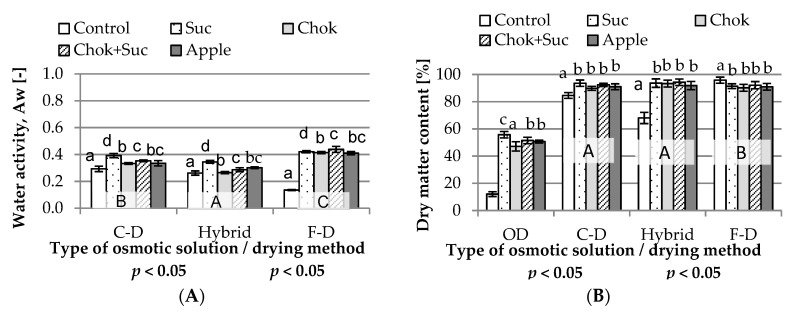
Influence of the type of osmotic solution on: water activity (**A**) and dry matter content (**B**) of osmo-dehydrated (at 50 °C for 24 h) and dried quince slices. Designations: A–C and a–d—homogeneous groups; Suc, Chok, Chok + Suc, apple—coding the type of osmotic solution, as given in the experimental procedure.

Drying of osmotically dehydrated quince slices resulted in an increase in the stability of chips by further reducing water activity (Figure 2A) and an increase in dry mater content (Figure 2B). In most dried quince, the water activity was 0.40 or lower. There was a significant effect of the type of osmotic medium (four homogenous groups; a–d) and drying method on the water activity of dried samples (three homogenous groups; A–C).

The highest values were found for freeze-dried samples, followed by lower values for convection-dried and then hybrid-dried samples. In addition, the lowest water activity occurred in dried samples without osmotic pre-treatment. Among all samples, freeze-dried controls (without osmotic treatment) had the lowest water activity (0.140–0.290). Quince fruit osmotically dehydrated in a sucrose solution and dried using a hybrid method showed less reduction in the water activity of the samples compared to the material dehydrated in the other solutions. Janowicz et al. [34] reported similar water activity (0.390–0.395) for apples dehydrated in sucrose and dried by convection, which is comparable to quince chips that were subjected to similar osmotic treatments and dried using a hybrid method (convective microwave and vacuum drying; 0.392). Similarly, Piotrowski et al. [35] showed that water activity of osmotically dehydrated and freeze-dried strawberries was in the range of 0.163–0.250, but without initial osmotic treatment, the water activity was 0.135–0.293. Hybrid drying of samples that were pre-osmotically dehydrated was more effective (lower microwave power, shorter time) compared to fresh samples without treatment. With the F-D method, the opposite was observed—OD complicated drying. The dried fruit had a less delicate texture (porosity) and was chewy, requiring longer drying times. OD before freeze-drying negatively affected on the process. Substances, especially sugars, that penetrate the material during OD hamper freeze-drying. Similar observations were reported by Janowicz et al. [36] who showed that dried fruit subjected to earlier OD differed from control samples; they were viscoelastic rather than delicate and fragile due to other water activity. Higher water activity obtained by F-D may be associated with the lack of crispness. According to Kondratowicz et al. [37], dried fruit obtained by F-D without preliminary dehydration are characterized by much higher quality than those subjected to traditional (convection) drying. They retain their color and volume (no shrinkage) and have an attractive texture. Under these conditions, adverse changes associated with glassy transition may occur: the internal structure of the dried material may be broken and freezing separates the aqueous solution contained in the product into a mixture of two phases, i.e., ice crystals and concentrated solution.

The dry matter content, as in the case of osmotically dehydrated fruit, was only influenced by the use of osmotic pre-treatment, but the type of osmotic medium had no significant effect on this indicator in dried samples. Moreover, the freeze-dried samples were characterized by a much higher dry matter content than those prepared using the other method. The samples dried with the hybrid method without pre-osmotic treatment were the most difficult to dry; their dry matter content was the lowest (68.0%).

The structure collapses when the matrix viscosity becomes very low due to too high a temperature; the structure loses its rigidity and the pores close before the drying process is completed. Conducting the process below the glass transition temperature ensures the stability of the structure of the dried material [24].

#### 2.2.2. Texture

**Figure 3:**
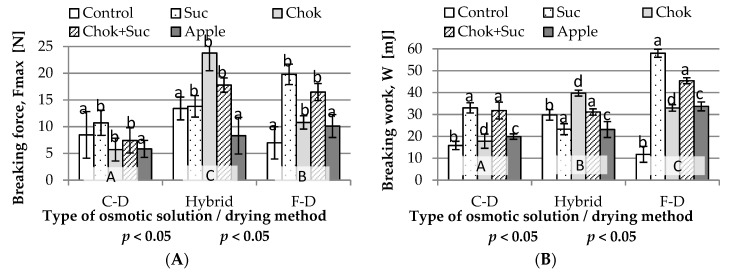
Influence of the type of osmotic solution and drying method on: (**A**) breaking force and (**B**) breaking work of quince chips. Designations: a–d and A–C—homogeneous groups; Suc, Chok, Chok + Suc, apple—coding the type of osmotic solution and C-D, hybrid, F-D—coding the type of drying method as given in the experimental procedure.

Mechanical and acoustical instrumental tests are useful for assessing the properties of dried food [38]. The breaking test was used in this study to assess the texture of quince chips. Hardness (maximum breaking force, Fmax) and breaking work (W) are important indicators in the evaluation of dried snacks (chips). They allow the determination of consumption usefulness. The obtained values were diverse (Figure 3A,B). The statistical analysis showed the influence of the type of osmotic solution and the drying method on the force and work needed to break the dried samples in the breaking test. Initial OD caused quince chips to become harder than these produced by omitting osmotic pre-treatment. Control samples (without osmo-treatment) and osmo-dehydrated in apple juice concentrate (one homogeneous group a) were characterized by breaking forces of 7.0–13.4 N and 5.8–10.1 N, respectively, and values of 7.4–23.8 N when dehydrated in other solutions (group b) (Figure 3A). Hybrid drying of quince resulted in the highest force values (average: 15.4 N) and the lowest values were produced using the convection method (average: 7.26 N). In the case of convective drying, the highest force was observed in the chips dehydrated in a sucrose solution (Suc) and mixture (chokeberry juice (Chok) + Suc). The value was similar with the use of the F-D method, although the values were about two times higher. With hybrid drying, the highest maximal force values were found in samples dehydrated in solutions containing chokeberry juice concentrate (Chok and Chok + Suc).

The drying method in combination with the type of solution used for pre-treatment (interactions) probably influenced the diversity of the obtained values. In addition, large standard deviations of the indicator were observed. Ciurzyńska and Lenart [39] found no relationship between the type of osmotic substance and the mechanical properties of osmotically dehydrated and then freeze-dried strawberries. In these studies, the use of apple juice concentrate for initial OD resulted in obtaining dried quince with a structure similar in hardness (homogeneous group a) to the control samples, which are the most desirable, especially in the production of freeze-dried fruit. Kozak [40] studied selected texture parameters of popular dried fruit and vegetable snacks available on the market. The force values obtained during the breaking test were in the range of 11.9–35.4 N. For carrot chips, the values were the closest to that of the breaking force of the quince chips, which was in the range of 5.7 to 23.8 N (Figure 3A). A carrot snack (at a head speed of 10 mm/s) had the softest structure and required the least force (approx. 22.0 N). In this study, dried quince produced by hybrid with preliminary dehydration in a solution of sucrose (23.3 N) and chokeberry juice concentrate (23.8 N) was the hardest among those tested (but at a head speed of 1 mm/s).

The work performed on breaking dehydrated and non-dehydrated samples was more diverse than it was with the breaking force (Figure 2B). Basically, due to the lowest breaking work values, the most brittle (crunchy) were freeze-dried samples without pre-osmotic treatment (11.8 mJ), and those dried using the same method, but after initial OD in sucrose solution, they were the hardest to break (58.0 mJ). Generally, samples dehydrated in sucrose solution or with its addition to chokeberry concentrate and dried by convection (31.8–33.0 mJ) and F-D (45.4–58.0 mJ) were characterized by the highest values of breaking work. This rule did not work in hybrid drying. In this case, the highest values were found for chips dehydrated in chokeberry juice concentrate (39.7 mJ). F-D of samples previously dehydrated in sucrose solution were characterized by about 5-fold higher value (about 58.0 mJ) of the breaking work in comparison with the value of samples without osmotic pre-treatment (about 11.8 mJ). Usually, samples dried without preliminary osmotic treatment are characterized by a crispy, but also a delicate structure. The penetration of sugars during preliminary OD adversely affects the F-D; the samples become less crunchy and delicate and chewy [29].

In the study by Changrue et al. [41] it was found that pre-osmotic dehydration of carrots in a sugar and salt solution strengthened the cell structure during microwave-vacuum drying. According to Janowicz, Litwińska, and Lenart [36] dried fruit samples subjected to osmotic pre-treatment resemble viscoelastic bodies but those dried without pre-treatment were more brittle and, consequently, less compressive force was needed to destroy their structure. Whereas Kowalska, Marzec, Kowalska, Trych, Masiarz, and Lenart [29] showed that unlike F-D, osmotic pre-treatment of strawberries positively affects the puffing method drying process and produces high-quality dried products. This is due to the phase transformation of the increased content of sugars, which during this process pass into the glassy state. This condition affects the stability of dried products. Choo et al. [42] demonstrated the usefulness of both hybrid drying methods (with and without pre-convection drying), which effectively reduced the specific-energy consumption during drying of Murraya koenigii leaves. Many other researchers have shown that, due to the penetration of the osmotic substance into the plant tissue, OD has a large impact on the structure and properties of products obtained by using microwave drying [41,43,44,45].

#### 2.2.3. Acoustic Properties

**Figure 4:**
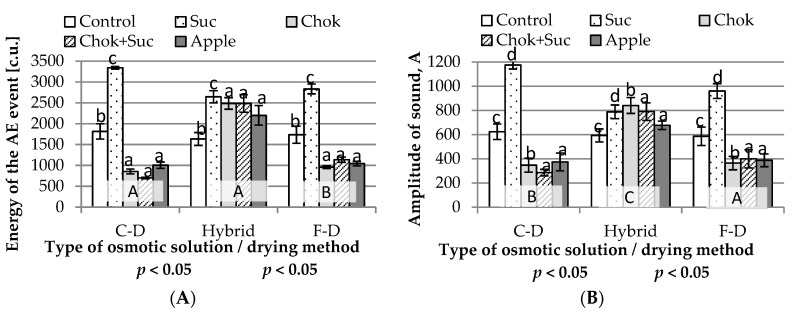
Influence of the type of osmotic solution and drying method on: (**A**) energy of the acoustic emission (AE) event and (**B**) amplitude of sounds of quince chips. Designations: a–d and A–C—homogeneous groups; Suc, Chok, Chok + Suc, Apple—coding the type of osmotic solution and C-D, Hybrid, F-D—coding the type of drying method as given in the experimental procedure.

**Figure 5:**
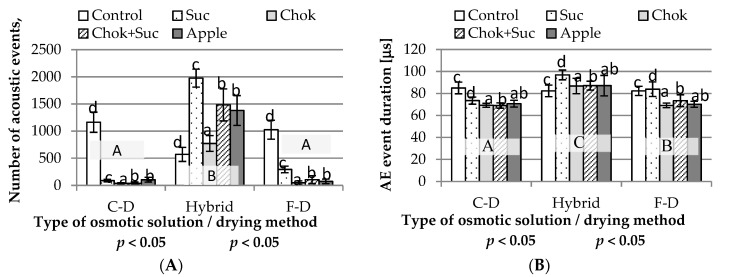
Influence of the type of osmotic solution and drying method on: (**A**) number of acoustic events and (**B**) AE event duration in breaking test of quince chips. Designations: a–d and A–C—homogeneous groups; Suc, Chok, Chok + Suc, apple—coding the type of osmotic solution and C-D, Hybrid, F-D—coding the type of drying method as given in the experimental procedure.

The chip sounds during the breaking test were determined by the energy of the acoustic emission (AE) events (Figure 4A), the amplitude of sound (Figure 4B), and the acoustic events (Figure 5A) and AE event duration (Figure 5B), reflecting their hardness and crispness. Statistical analysis showed that the type of osmotic medium and drying method had a significant effect on the AE of the chips.

The highest-energy AE event values were found in chips dehydrated in sucrose solution, regardless of the drying method (2645.4–3339.0 e.u.; homogeneous group c) (Figure 4A). The control samples showed less similar values of the AE event (1633.8–1815.5 e.u.; homogeneous group b). The samples dehydrated in sucrose solution and dried using all three methods showed the highest values of water activity compared to other samples (Figure 2A), and most of them also showed the highest energy of the AE event (Figure 4A). In the convective- and freeze-dried samples (homogeneous group B) (except for the control and the samples dehydrated in sucrose solution), pre-dehydrated samples showed up to a two-fold lower energy in the AE event value (Figure 4A). This probably caused them to become more delicate, crispier and less noisy than other chips that were harder. More similar values of the AE event were for hybrid-dried samples (1633.8–2645.4 e.u.); they were quite hard.

The amplitude of sound recorded during the chip breaking test was also quite diverse (Figure 4B), but the effect was similar to the energy of the AE event (Figure 4A). Specific differences on amplitude of sound were noted between samples dried using hybrid method (Figure 4B). These samples were the least differentiated, having relatively high values in the range of 593–840 mV in control chips and obtained by drying after osmo-dehydration in chokeberry juice concentrate. Chips produced by dehydration in sucrose solution and convection (1175 mV) or F-D (960 mV) produced the highest sound amplitudes. In contrast, the lowest amplitude of sound was recorded in chips osmotically pre-treated in chokeberry juice concentrate or mixed with sucrose solution and in apple juice concentrate, which were dried by C-D and F-D (285–400 mV).

Marzec et al. [46] in their work on various methods of drying cherries found that freeze-dried samples had the lowest amplitude of sound. Some variants of freeze-dried quince fruit were characterized by values similar to those for cherries dried by the same method. The analysis of the amplitude of sound, similar to the energy of the AE event, showed significantly higher values of this indicator for osmotically dehydrated and dried hybrid chips compared to control samples (without pre-osmotic treatment). The above results may be related to a lower WL/SG osmotic dehydration index in the sucrose solution, which could have caused the lowest water loss in relation to the solids gain (Figure 1A), leading to a significant change in the structure of the dried samples.

During the breaking test, the hardness of the chips was determined by means of an acoustic discriminant, i.e., the number of acoustic events (Figure 5A). Statistical analysis showed a significant relationship between the type of osmotic solution and the drying method with the number of acoustic events recorded during breaking testing of quince chips. Samples osmotically pre-treated and dried using the hybrid method were characterized by the highest (570 to 1976) number of acoustic events, whereas the values of this indicator for the same samples but dried by the two other methods were significantly lower, mainly from 37 to 106, and chips dehydrated in sucrose solution and freeze-dried had 294 events. In addition, the number events in the control samples obtained by the hybrid method were about two times lower than that of the other control samples.

In most cases, quince dried by convection and F-D had significantly higher water activity (Figure 2A) and lower numbers of acoustic emission events. Moreover, Marzec, Kowalska, and Oldak [46] determined that the number of events during the compression test of dried cherries was the highest of the samples produced by the hybrid method. Similarly, in the crackers quality test [47] using the method of acoustic emission, with increasing water activity, the acoustic events decreased due to the impact of different stress distribution in dry and moist materials.

The AE duration in the breaking testing of the chips varied in the range of 70–95 µs (Figure 5B). A significant impact of the drying method and the osmotic type of solution on the time of sound emission was found. The duration of the acoustic emission events recorded during breaking testing of quince chips was homogenous in three groups in terms of drying method and four groups in terms of type of solution (Figure 5B). Hybrid drying produced the highest values of the indicator, significantly lower in the case of F-D, and the lowest was found in convective dried chips.

#### 2.2.4. Color Parameters

**Figure 6:**
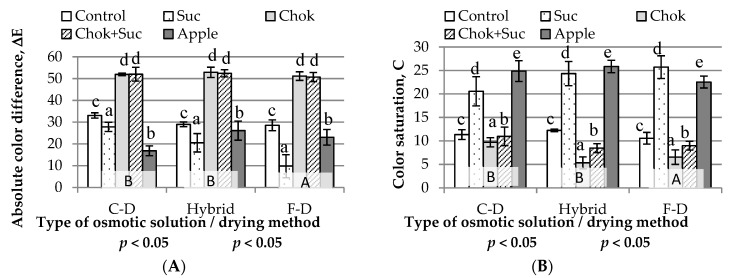
Influence of the type of osmotic solution and drying method on: (**A**) absolute color difference and (**B**) color saturation of quince chips. Designations: a–d, A, B—homogeneous groups; Suc, Chok, Chok + Suc, apple—coding the type of osmotic solution and C-D, hybrid, F-D—coding the type of drying method as given in the Experimental procedures.

Analyzing the absolute color difference, the color of all chips significantly varied from the color of the raw material; the values ranged from 10 to 53. The type of osmotic solution used for the initial dehydration and drying method significantly affected the color of the dried fruit (Figure 6A). Regardless of the drying method, dehydration of quince fruit in sucrose solution (10–28) and apple concentrate (17–26) caused the least color changes; however, they changes could be clearly perceived by the human eye. Chips obtained as a result of preliminary dehydration in a sucrose solution looked attractive and those dehydrated in a chokeberry juice concentrate were very dark in color. The chips produced using the convection method were distinguished by visible browning. Freeze-dried quince samples were significantly different from those obtained by other methods, with a mainly lower color difference compared to the raw material.

High values of absolute color difference were also found in the control samples (without osmotic pre-treatment; 29–33). A particularly visible change in color in relation to the raw material occurred in the case of osmotically quince slices dehydrated in chokeberry juice concentrate and in a mixture of this concentrate with a sucrose solution (51–53). Chokeberry juice concentrate, due to the content of colorful anthocyanin compounds, caused intense coloration combined with darkening of the sample color. As a result, it was impossible to distinguish chips pre-dehydrated in chokeberry juice concentrate with those dried by various techniques.

At any stage of quince fruit chip production, color changes may occur, both during OD and when drying by various methods. Maillard reactions and caramelization of sugars may occur during the drying process of plant materials at higher temperatures [48]. These phenomena are classified as non-enzymatic browning reactions. Both processes are responsible for the external appearance, taste, and aroma of the finished product. During F-D, the slightest color changes occurred compared to the raw material. F-D is usually carried out at low temperature, which is why sugars are not caramelized. According to Zielinska, Ropelewska, Xiao, Mujumdar, and Law [45], the initial osmotic treatment has a positive effect on the color of fruits subjected to microwave-vacuum drying. They explained this by the shortening of the drying time compared to other drying methods. As a result, the pre-treatment increases the drying rate and shortens the time for oxidative browning reactions.

The color saturation of the quince chips was significantly different for the different types of osmotic solution and drying methods (Figure 6B). This color parameter was largely influenced by initial OD in sucrose solution and apple juice concentrate. Regardless of the drying method, the color saturation of these dried quince fruit was at least two-fold higher compared to those without osmotic pre-treatment and five-fold higher compared to the color of other chips.

#### 2.2.5. Sensory Evaluation

**Figure 7:**
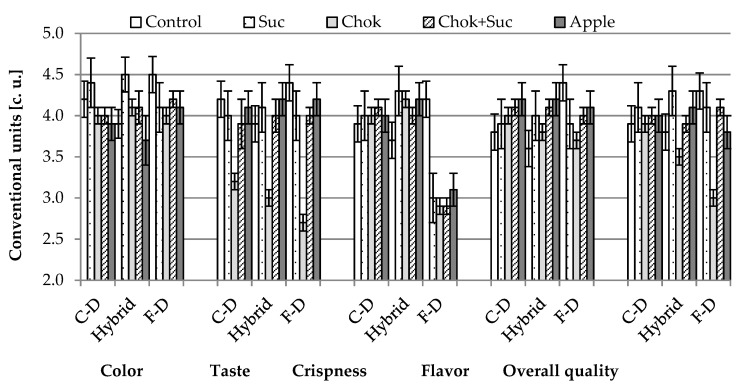
Influence of the type of osmotic solution and drying method on sensory properties of quince fruit chips—coding the type of drying method as given in the experimental procedures.

According to respondents, all chips obtained using the studied osmotic solutions and drying methods were highly rated (Figure 7). Considering the sensory attributes of samples osmotically pre-treated using different osmotic solutions, chip evaluation ranged from 3.8 (dehydrated in chokeberry juice concentrate) to 4.1 (in sucrose), including the control sample, which was rated 4.0 The mean scores for color (4.1) and taste (4.0) were the highest; scores were lower in terms of taste (3.8) and crunchiness (3.9). Color is one of the most important and widely used factors in the food industry, which is directly related to the quality of the products [49]. The color of hybrid and convection chips (4.4–4.5) obtained by initial dehydration in sucrose solution and then F-D (4.5) without osmotic treatment was rated the highest.

The crispness of the hybrid chips was assessed at 4.0–4.3 but 2.9–3.1 when osmo-dried by F-D, and 4.0–4.1 when dried with the convection method. The crispness of the control hybrid chips was assessed at 3.7, but significantly higher, 4.2, by the F-D method (delicately crisp and crunchy samples). This confirms previous research [29] in that unlike F-D, osmotic pre-treatment of strawberries positively affects the puffing method drying process and produces high-quality dried products. Despite the long F-D time (24 h), these chips may not have been dried sufficiently or the initial OD caused a structure change and adversely affected the crispness. Taste also differentiated the quality of the chips. Regardless of the drying method, the samples initially dehydrated in the chokeberry juice concentrate were assessed the lowest (2.7–3.2), and the highest were those produced as control (3.9–4.4) and using dehydration in sucrose solution (4.0–4.2 or apple juice concentrate (4.1–4.2). Samples dehydrated in a solution consisting of sucrose and chokeberry juice concentrate were also highly rated (3.9–4.0); they beneficially complemented taste by the mix of sweet, sour, and tart substances. It can be concluded that both crisp texture with a natural color and a strong dark-red color were accepted. Ciurzyńska et al. [24] found that, in comparison to other drying methods, F-D allows obtaining dried products with preserved color, both after and without pre-osmotic treatment. According to Zielinska, Ropelewska, Xiao, Mujumdar, and Law [45], pre-osmotic treatment improves the taste, color, and mechanical resistance of products dried by microwave convection, mainly due to the increased sugar content.

**Figure A1:**
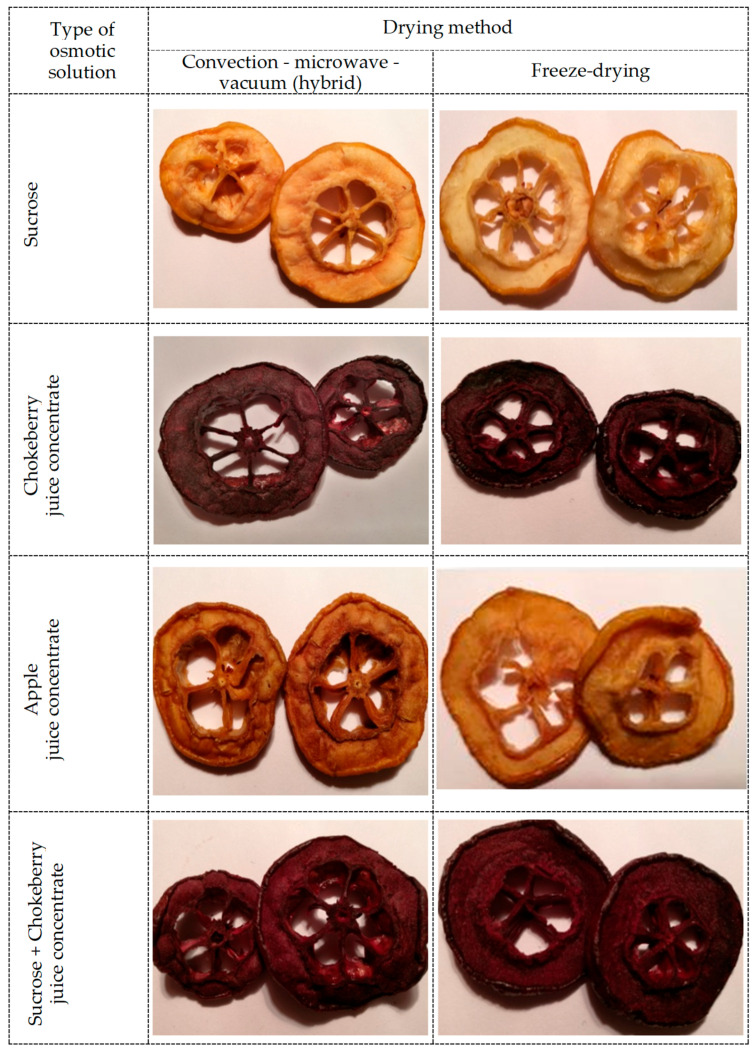
Pictures of Japanese quince chips pre-osmotically dehydrated and dried; slices of maximum and minimum diameters, cut from fruit.

Characteristic blisters were observed on the surface of the chips produced by hybrid drying. In addition, respondents’ opinions showed that slices from the desirable part of the Japanese quince fruit that had the largest diameter (Figure A1, Appendix A) were more desirable in terms of appearance. These slices obtained through the cross-section of the fruit also contain a cross-section of the seed chamber. Seed nest shells separating the seeds from each other form the shape of a characteristic star, which is more visible in larger diameter slices, hence, the respondents’ opinion on the higher attractiveness of Japanese quince slices with larger diameter.

Particular attention should be paid to the possibility of using juice concentrates as factors shaping the sensory quality. Their price can compete with chemically produced dyes or other substances for flavor and flavor development. The processed fruit gains additional compounds with health-promoting properties.

#### 2.2.6. Principal Component Analysis (PCA)

**Figure 8:**
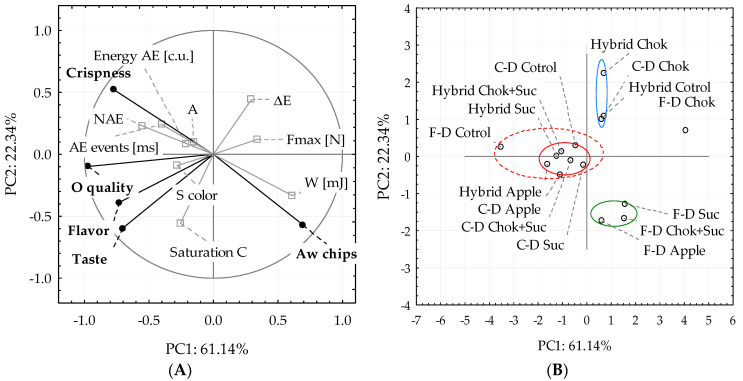
Principal component analysis (PCA) analysis: (**A**) PCA diagram; (**B**) similarities and differences. Meanings: O quality—overall quality, S color—color sensory evaluation, saturation C—color saturation parameter.

To detect similarities and differences (correlations) between the analyzed quince chips in terms of initial osmotic treatment and properties evaluated in terms of physical and sensory properties, principal component analysis was performed (Figure 8A). The first two principal components PC1 (sensory discriminants, water activity of chips) and PC2 (other indicators) explained 83.48% of the variability of the properties of dried fruit (Figure 8A,B). The first principal component (61.14% of the total variance) was highly correlated with water activity and work of breaking, in lesser degree with absolute color difference and force of breaking while it was negatively correlated with all sensory discriminants.

The second principal component (22.34% of the total variance) was highly correlated with crispness, number of acoustic events, color difference and in minor degree with other acoustic and indicators while it was negatively correlated with water activity, work of breaking and other sensory discriminants.

It was observed that chip crunchiness was slightly related to the acoustic properties; only with number of acoustic events (NAE), the correlation was statistically significant. This discriminant was also positively correlated with overall quality. The crispness of chips was inversely proportional to the water activity and breaking work (mechanical properties). Water activity is an important determinant of the crispiness of dried foods [50]. The acoustic events are an important indicator of the quality of the food texture, especially of the snack physical properties, reflecting the consumer’s assessment of quality [51]. However, as concluded above in Section 2.2.2 (Figure 3A), the large standard deviations in the values of the instrumental indicators due to the lack of a uniform shape of the quince chips (wavy surface, blisters, with or without a specific seed box) resulted was quite varied.

Although the dark red color of the chips obtained by preliminary dehydration in the chokeberry juice concentrate was positively assessed, the greater the color differences (absolute color difference) (too dark, deviating from the colors characterizing the color of the raw materials), the lower the sensory evaluation. The use of chokeberry juice concentrate resulted in a poorly accepted taste and too large differences in the color of the quince chips (significant darkening), which lowered the color saturation values of the samples. The use of the chokeberry juice concentrate and sucrose mixture improved the taste of the chips and reduced the negative sensory feelings resulting from too much of the chokeberry concentrate components (tannins and polyphenols) that penetrated the pre-treated samples.

The use of pre-osmotic dehydration with a different type of osmotic solution, including juice concentrates, and the method of drying enabled the division of the obtained data, as shown in Figure 8B. A separate group (green line, Figure 8B) included freeze-dried chips after osmotic pre-treatment in sucrose solution, mixture chokeberry concentrates, and sucrose, and in apple concentrate. These dried quince chips received lower sensory scores (Figure 7), especially crunchiness. These samples may have been rubberier. The value of AE, number of events, and amplitude of sound were similar to most samples pre-osmotically dehydrated and dried by the convection method. On the other hand, these chips showed higher values of compression work compared to those produced by other drying methods. Dried samples obtained by osmo-dehydration in chokeberry concentrate and dried regardless of the method and that the control hybrid constituted the group marked with the blue line, mainly due to the lower-rated taste and quality of hybrid control samples. Another group consisted of all the other samples (dashed red line) (Figure 8B), mainly hybrid or convective-dried chips after preliminary dehydration into sucrose solution, apple concentrate and mixture of sucrose and chokeberry juice concentrate or without the F-D control (red line). This division was performed by considering the sensory evaluation.

From the above considerations, both juice concentrates can be used as osmotic agents for pretreatment and the tested drying methods can be used to produce high quality quince snacks. The processes for the production of such snacks should be chosen primarily on the basis of process efficiency and economic aspects, as well as sensory quality. Other researchers [52] showed that drying at reduced pressure with simultaneous microwave heating allows to obtain a product with very good properties. By microwaves, heat is supplied to the entire volume of the material in a very short time; therefore, the heat transfer of the dried material is more effective compared to traditional drying methods. The reduced pressure during the process reduces the boiling point of water in relation to the atmospheric pressure and significantly reduces the adverse thermal effect on the nutritional value and sensory properties of the product.

The use of juice concentrates should be treated as an important factor, which allows the sensory quality of snacks to be shaped, especially that of the unpopular quince chips. The importance and need for a wider use of juice concentrates and quince fruit, as shown in many publications [6,8,9,10,15], refers to the richness of their natural compounds with health-promoting properties. The combination of C-D and microwave-vacuum drying (hybrid method) provides a viable alternative to F-D, which is recognized as the best in terms of quality and maintaining the nutritional value of products. The use of microwave-vacuum heating significantly shortens the drying time and enables obtaining high-quality dried material [52].

## 3. Materials and Methods

### 3.1. Materials

For testing, firm and ripe similar-sized (4.5 ± 0.3) cm diameter fruit of Japanese quince (Chaenomeles Japonica (Grójec, Poland), bought directly from the orchardman, was used. The fruit was washed and cut into slices about 3 mm thick in a Robot Coupe chopper (CL50 STALGAST 713500, Stalgast Ltd., Radom, Poland) with the skin, but the seeds were removed. Osmotic solutions were sucrose (Polish sugar) with a concentration of 70 °Bx (Suc) and juice concentrates (Białuty Company, Radzików, Poland): chokeberry (Chok) with a concentration of about 65.1 °Bx and apple (Apple, Białuty Company, Radzików, Poland) with concentration of about 70.3 °Bx. A mixture of chokeberry juice concentrate and sucrose solution (Chok + Suc) in a 1:1 ratio was also used. Osmotic solutions from fruit juice concentrates were prepared by diluting them with distilled water until obtaining water activity at 0.81 ± 0.02.

### 3.2. Experimental Procedure

The quince slices were subjected to OD in concentrates of chokeberry and apple juices and a mixture of concentrate with sucrose, and then dried using one of the following methods (Table 1). Samples that were not subjected to osmotic pre-treatment (Control) were also dried. Drying was performed in 2 repetitions, and OD before each drying was performed in parallel in 3 or more replications to obtain the appropriate amount of dried material for the individual physical and sensory determinations.

### 3.3. Osmotic Pre-Treatment

The samples (about 150 g each) were osmotically dehydrated in beakers, which were placed in a water bath (JW ELECTRONIC type T-OSM, Warsaw, Poland) with shaking at a frequency of 1 Hz at a constant temperature of 50 °C for 24 h. After dehydration, the samples were rinsed under running cold water and dried with blotting paper. The ratio of the solution weight to the fruit weight was 4:1.

### 3.4. Drying

#### 3.4.1. Convection Drying (C-D)

Convection drying was carried out in a laboratory belt dryer with parallel airflow at a speed of 1.5 m/s with a belt travel of 1 mm/s. The process temperature was (60 ± 2) °C and the time was 5 h. The quince slices were evenly distributed in one layer.

#### 3.4.2. Hybrid Drying (Hybrid)

The hybrid drying method consisted of C-D in a forced air dryer at 60 °C and a speed of 1.5 m/s for about 1 h, then placement in a PROMIS µLAB (Wrocław, Poland) microwave-vacuum dryer. This drying consisted of 4 stages, and their total time was about 10 min. Constant values of drying parameters were used: duration of about 10 min, microwave power 200 W, pressure 3.5 kPa, and maximum steam temperature of 60 °C.

#### 3.4.3. Freeze-Drying (F-D)

Before F-D, the samples were frozen in a shock freezer with air at –40 °C for 4 h and then dried in an ALPHA 1-4 Martin Christ Gefriertrocknungsanlagen (Osterode am Harz, Germany) freeze dryer at 20 °C with a shelf pressure of 63 Pa for 24 h.

### 3.5. Analytical Methods

The dry matter content and water activity, mechanical, and acoustic properties (crisp structure), and color parameters were calculated for quince chips.

OD was characterized on the basis of an index calculated as the quotient of water loss (WL; g H_2_O/g initial dry matter (i.d.m.)) and solid gain (SL; g/g i.d.m.) in relation to the i.d.m. content [5,39].

#### 3.5.1. Determination of Dry Matter Content

The dry matter content of each tested sample (fresh, osmo-dehydrated, and dried) was determined gravimetrically (AOAC 920.15, 89 2002) by vacuum drying (SPT-200, ZEAMIL HORYZONT, Krakow, Poland) at ≤100 mmHg and a temperature of 70 °C until a constant weight was achieved, which was about 24 h. The samples were weighed using an analytical scale with an accuracy of 0.001 g. The measurements were performed twice.

#### 3.5.2. Determination of Water Activity

Water activity (Aw) in the raw material and samples subjected to OD was measured using an AquaLab 3 TE meter (Decagon Devices Inc. Pullman, WA, USA) at 25 ± 1 °C. The measurements were recorded in triplicate.

#### 3.5.3. Examination of Crisp Structure

The mechanical properties of quince snacks were tested using a Texture Analyzer TA-HD plus texture meter (Stable Micro Systems, Godalming, UK) with a 5 kg measuring head. The tested material was subjected to a breaking test by placing it on supports spaced 20 mm wide. Single quince chips of similar diameter in the range of (38 ± 2) mm were tested. Prior to testing, after production, the dried quince chips were stored at room temperature (about 23 °C) for about 24 h in a sealed package, protected from light. The breaking test was carried out at a constant deformation speed of 1 mm/s. The measurement was performed in 10 replications. Selected mechanical features, such as maximum force (hardness) and breaking work, were measured.

Breaking work (W, (mJ)) was calculated by multiplying the area under the curve in the force–time curve with the speed of the head:

W= v·dF/dt(1)

where W is the breaking work (mJ), v is the head speed (mm/s), and dF/dt is the area under the curve of the force-time curve (N·s).

During the breaking test, the sound of broken (cracking) chips was recorded. The acoustic emission (AE) was registered with a contact method using the 4381 sensor (Brüel; Kjær, Naerum, Denmark) in a breaking test. The AE was recorded in the frequency of 1–15 kHz. The obtained signal was amplified (AED = 3) and recorded using a program cooperating with the Texture Exponent 32 texturometer (Stable Micro Systems). Four different parameters characterizing AE were determined: energy of the AE event (contractual unit; (j.u.)), number of acoustic events (NAE), amplitude of sound (A; (mV)), and AE event duration (μs), as previously defined [50,53].

#### 3.5.4. Color Parameters

The color parameters of raw slices of quince and chips were measured with the help of Chroma Meter CR-300 from Konica Minolta (CIE standard observer 2°, illuminatus D65, measuring aperture 8 mm, Osaka, Japan) The color was examined in CIE Lab space. The color of each sample arranged in layers was measured in 5 replications at random locations.

The absolute color difference (Δ*E*) and the color saturation (*C*) were calculated:

(2) $$\Delta E=\sqrt{\Delta L^{2}+\Delta a^{2}+\Delta b^{2}}$$

(3) $$C=\sqrt{a^{2}+b^{2}}$$

where *L* is the brightness of color, *a* is chromaticity in the red (+*a*)–green (–*a*) range, *b* is chromaticity in the yellow (+*b*)–blue (–*b*) range, and ∆*L*, ∆*a*, and ∆*b* are the surface color difference indicators in relation to the color of raw material samples.

### 3.6. Sensory Evaluation

A sensory evaluation of dried quince samples was conducted in laboratory conditions by a team of 30 people (22 women and 8 men, all between 22 and 26 years of age). Prior to testing, a training session was held to familiarize the panelists with the products and selected attributes, as described in literature data [54,55]. The consumer evaluation was carried out in a laboratory with separate individual evaluation points, in the same, constant conditions of temperature and humidity, in accordance with the ISO standard [54]. The samples were marked with 2-digit random numbers in random order and presented on white plastic plates. Water (room temperature) was recommended as a neutralizing agent between evaluations of different samples. The panelists assessed the dried quince samples and marked the results on a hedonic scale ranging from 0 to 5, with specific boundary conditions: 0—unacceptable; 1—moderately unacceptable, 2—neutral; 3—moderately acceptable; 4—very acceptable; 5—satisfactory. The following discriminants were prepared for the evaluation of the selected qualitative characteristics of the analyzed samples: color (dark—0; slightly dark yellow or red as a result of osmotic pre-treatment in chokeberry juice concentrate—5); taste (sour, bitter—0; slightly sweet and sour, slightly bitter—5); crunchiness (quiet—0; loud—5), overall quality (bad: 0; very good—5). The results of the sensory evaluation are expressed in conventional units; c.u.

### 3.7. Statistical Analysis

The obtained results (average values) were subjected to statistical analysis in the Statistica12 program (vol. 12, StatSoft PL). One- and two-factor ANOVA and Tukey’s test were performed to demonstrate the effect of the type of osmotic solution and drying method on the selected indicators and physical properties of osmotically dehydrated and dried samples. Statistical calculations were carried out at the α = 0.05 level. Data groups that were not statistically different were assigned to the same homogeneous group. To evaluate the dependence of the sensory assessment on the results of experimental studies (WL/SG, depending on type of osmotic medium; Aw of dried quince; mechanical with acoustic properties) and to determine the relationship between the selected features, matrix correlation analysis, and principal component analysis (PCA) were performed.

## 4. Conclusions

The use of OD of Japanese quince fruit in juice concentrates and sucrose solution and drying methods produced the required reduction in water, ensuring microbiological stability. The type of osmotic solution and drying method significantly impacted the tested mechanical and acoustic properties of dried quince samples. Most of the osmotically pre-treated chips had higher force and breaking work valued compared to non-dehydrated samples. Hybrid drying predominantly produced the highest values of these indicators. The quince chips that were dehydrated in the first stage of their production in a solution consisting of sucrose and chokeberry juice concentrate, and then dried using the hybrid method, were highly rated. Crunchiness as one of the most important sensory attributes of dried food was highly correlated with the overall quality of quince chips, their water activity, and the number of acoustic events recorded during the breaking test. The use of sensory evaluation can be useful in determining the quality of the chips in terms of their overall quality and texture (crispness), which can be replaced by instrumental methods (breaking tests with acoustic recording using PCA.

Hybrid drying, consisting of convection and microwave-vacuum drying, is a useful method for the production of quince fruit snacks.

Further research is planned to demonstrate the usefulness of fruit juice concentrates and microwave-vacuum convection drying for larger-scale production of quince fruit snacks. Therefore, detailed analysis of the chemical composition of such snacks is also important in terms of preserving natural bioactive compounds and antioxidant activity.

## Figures and Tables

**Table 1:** Summary of sample codes.

Type of Treatment			
Type of Samples/Type of Osmotic Solution	Coding	Drying Method	Coding
Raw material	Fresh	Dried without pre-treatment	Control
Pre-osmotic dehydrated in sucrose solution	Suc	Convection	C-D
Pre-osmotic dehydrated in chokeberry fruit concentrate	Chok	Convection and microwave-vacuum	Hybrid
Pre-osmotic dehydrated in mixture of chokeberry juice concentrate and sucrose	Chok + Suc	Freeze-drying	F-D
Pre-osmotic dehydrated in apple juice concentrate	Apple		

## Abbreviations

WL	water loss (g/g i.d.m.)
SG	solid gain (g/g i.d.m.)
i.d.m.	initial dry matter content (g)
WL/SG	dehydration efficiency
Suc, Chok Chok + Suc Apple	coding the type of osmotic solution; respectively: sucrose solution, chokeberry juice concentrate, chokeberry juice concentrate and sucrose solution mixture, apple juice concentrate
C-D, Hybrid F-D	coding the drying method; respectively: convective drying, hybrid drying (convective and next microwave-vacuum drying), freeze-drying
Control	fruit samples dried without pre-osmotic treatment
NAE	Number of acoustic events
